# Willingness to pay for a National Health Insurance (NHI) in Saudi Arabia: a cross-sectional study

**DOI:** 10.1186/s12889-022-13353-z

**Published:** 2022-05-12

**Authors:** Abeer Alharbi

**Affiliations:** Health Administration Department, Business Administration College, King Saud University, Riyadh, Saudi Arabia

**Keywords:** Health insurance, National health insurance, Health system, Willingness to pay, Reform, Health care delivery, Saudi Arabia

## Abstract

**Background:**

Healthcare services in Saudi Arabia are provided free of charge to its citizens at the point of use. Recently, however, the government has realized that this model is unsustainable in the long run. Therefore, Saudi decision-makers are seeking to have a sustainable health system through the introduction of a contributory National Health Insurance that require making regular financial contributions from its members.

**Objective:**

This study aims to explore the people’s willingness to pay for a National Health Insurance system in Saudi Arabia. The study also aims to understand the factors affecting their willingness or unwillingness to pay NHI, such as, their demographic and socio-economic characteristics, the type of their usual health care provider, and their satisfaction with the current healthcare services.

**Methods:**

A cross-sectional study design with Contingent Valuation (CV) technique was used to measure the value of National Health Insurance based on an individual’s willingness to pay. The data were collected from 475 participants using an online survey via Google Forms between March 2021 and April 2021. Frequencies, logistic regression, and linear regression, were conducted to answer the research questions.

**Results:**

The number of individuals who was willing to pay for NHI was higher than those who were not willing to pay (62.9, 95% CI = 58.4-67.3%) vs (37.1, 95% CI = 32.7-41.6%). A binomial test found this difference was statistically significant (*p <* 0.001). There was a significant association between the likelihood of paying for NHI and type of usual healthcare provider (OR = 3.129, 95% CI = 1.943-5.039, *p <* 0.001); as individuals using public health services were more likely to pay for NHI. Also, with satisfaction with health services (OR = 14.305, 95% CI = 3.240-63.153, *p <* 0.001), as individuals who were very satisfied with the healthcare services were more likely to pay for NHI. The median amount of money the people were willing to pay as a monthly contribution for NHI was 100 SAR (26.5 USD) with the average being 152 SAR (40 USD). There was a significant association between the maximum amount the participants were willing to pay and age, region, and education. Specifically, 30–39-year-olds were willing to pay more for NHI compared to participants aged 50 or older (ß = 103.55, 95% CI = 26.27- 199.29); participants from central region more than participants from northern region (ß = 70.71,95% CI = 2.14- 138.58); and participants with masters degree more than participants with PhDs (ß = 227.46, 95% CI = 81.59- 399.28).

**Conclusion:**

This study provided some evidence that more people were willing to pay for NHI than those who declined. Individuals who frequently used public health services and were very satisfied with these services were more willing to pay for NHI. Younger population, those with master’s degree, and from the central region were willing to pay more amount of money for NHI. These results could help policy makers shape their decisions and anticipate problems that may arise with NHI implementation.

**Supplementary Information:**

The online version contains supplementary material available at 10.1186/s12889-022-13353-z.

## Background

Healthcare services in Saudi Arabia are provided free of charge to its citizens at the point of use, which may result in a huge financial burden on the government budget. These services are provided by the Ministry of Health’s (MoH) network of hospitals and primary healthcare centers through which health services are distributed throughout the country, and other governmental institutions (military or university hospitals). The government finances public healthcare services from the oil revenues without collecting contributions from the citizens. Health expenditure as a share of GDP for Saudi Arabia was 6.4% in 2020 [1, 2]. The budget for healthcare in 2021 was SAR 175 billion ($46.4 billion), an increase of 4.6% of the SAR 167 billion ($44.2 billion) budgeted in 2020 [2]. The government spending as a % of total healthcare expenditure accounts for the majority of the total healthcare expenditure and stands at 75%, where the share of private sector stands at 25% [3]. Healthcare costs are increasing globally, and Saudi Arabia is facing these same escalations. At present, the Saudi citizens population is estimated at 21 million, 6% of whom are aged 65 and above [4]. It is predicted that the older population will increase and make up 18.4% of the total population by 2050 [5]. While the rate of communicable diseases has decreased, that of non-communicable diseases, especially cardiovascular ones and diabetes, have increased [6]. Aging, change in disease pattern, and a rapid increase in population make the financing of healthcare an important and urgent issue. Moreover, the availability and quality of public healthcare services have been a persistent issue in the Saudi health system. Further, the free health care model has led to overutilization and longer waiting time for hospitals and specialists [7–10].

Despite reforms in the private health sector through the implementation of compulsory health insurance for expatriates, the Saudi health system still needs to mobilize additional resources to ensure the sustainability of the public healthcare services, especially with the fluctuations in oil prices. Saudi Vision 2030, an initiative established to reduce Saudi Arabia’s dependence on oil by diversifying its economy, also seeks to improve and develop all public services, including healthcare. The vision has placed financing reform as a primary transformational enabler for a universal healthcare coverage which will ensure that all citizens, residents, and visitors to Saudi Arabia can obtain timely access to healthcare services, via insurance, without the risk of impoverishment [11]. The transformation involves creating a broader role for private health insurers, through the creation of a market of licensed and regulated insurers who will offer National Health Insurance (NHI). The NHI is a contributory insurance scheme into which citizens are required to make regular contributions which would supplement the government’s health budget to meet the increasing costs. The contribution would be similar to an insurance premium with no refund for those who do not need to use the health services. The proposed model is similar to the private health insurance now being used for workers in the private sector. The NHI is expected to relieve some of the financial burden on the government as well as to improve the availability and quality of the public health services.

Little is known about the Saudi population’s willingness to pay (WTP) a regular contribution for this proposed National Health Insurance (NHI) scheme. Examining the Saudi population willingness to pay for the proposed NHI could help policy makers shape their decisions and anticipate problems that may arise with its implementation. Thus, this study set out to explore the people’s willingness to pay for a national health system in Saudi Arabia. The study also aims to understand the factors affecting their willingness or unwillingness to pay NHI, such as, their demographic and socio-economic characteristics, the type of their usual health care provider, and their satisfaction with the current healthcare services. The study also tries to assess the WTP for NHI at the national level to assist policy makers in determining the premiums in a more accurate way. Specifically, the study set out to answer the following research questions:

1. Are Saudi citizens willing to pay for a National Health Insurance (NHI)?
2. What is the maximum amount of money people are willing to pay for NHI?
3. Is there an association between the willingness to pay for NHI and the people’s demographic and socio-economic characteristics, their type of usual health care provider, and their satisfaction with the current healthcare services?
4. Is there an association between the maximum amount of money people are willing to pay for NHI and the demographic and socio-economic characteristics, their type of usual health care provider, and their satisfaction with the current healthcare services?
5. What are the reasons for any unwillingness to pay for NHI?

## Methods

### Study design

A cross-sectional study design with Contingent Valuation (CV) technique was used to measure the value of NHI based on an individual’s willingness to pay (WTP). WTP it is defined as the maximum amount that an individual is willing to pay for a good or a service. Typically, an individual’s WTP is measured by examining the price of the goods and services bought and sold in the marketplace. However, it is difficult to do the same for commodities which are not typically traded in the marketplace such as NHI. Therefore, this study resorted to the use of Contingent Valuation (CV) to investigate the willingness to pay for such an item. Contingent valuation (CV) surveys involve posing questions in such a way that the responses are contingent upon hypothetical markets described to the respondents. A number of previous studies have used CV methodology to examine the WTP for health insurance in developing countries [12–15], and two studies have been conducted in Saudi Arabia to assess WTP for NHI in one region of the country [16, 17].

### Data collection instrument

A questionnaire with two sections was used for this study. The first section presented a hypothetical scenario describing the implementation of a NHI that would ensure the sustainability of the current public healthcare services [Additional file 1]. Then, respondents were asked whether they would be willing to pay a monthly premium for this NHI scheme. If they declined to contribute, respondents were asked to give a reason. If they agreed to contribute, respondents were asked to state the maximum amount they would be willing to pay as a monthly insurance premium. The second section collected information on the respondents satisfaction with the public healthcare services rated on a 5-point Likert scale; type of health care provider (public or private); and their demographic and socio-economic characteristics, including their gender, age, marital status, region, education, income, and occupation.

### Sample size

The study population consisted of all Saudi individuals aged 18 years or older in Saudi Arabia. According to the statistics of the General authority of Statistics (GAS) on Saudi population, the total study population was approximately 14.5 million [18]. The sample size was calculated using a margin of error of ±5%, a confidence error of 95%, a 50% response distribution, and a population size of 21 million to arrive at the minimum required sample size of at least 385 participants. However, by employing the convenience sampling method, the study population were invited to participate in the study using an online survey via Google Forms between March 2021 and April 2021, of which 475 responded.

### Data analysis

The data were analyzed using SPSS version 23.0. Descriptive analysis was computed for the research variables and the demographic factors. A multiple binary logistic regression was used to determine the independent predictors for willingness to pay (WTP). All predictor variables were entered into the model simultaneously (no interaction terms were included), and all assumptions of homoscedasticity, singularity, and multicollinearity were met. The overall model test was significant, χ^2^(25) = 83.029, *p <* 0.001, and McFadden’s Pseudo R^2^ = 0.133. A multiple linear regression was used to determine associations of selected variables with the dependent variable which is the maximum amount people were willing to pay for NHI. The predictor variables selected for the models are gender, age, marital status, region, education, income, occupation, type of healthcare provider, and satisfaction with current public or private healthcare services. All predictor variables were entered into the model simultaneously (no interaction terms were included). For this analysis, as the dependent variable (maximum amount of monthly contribution) was highly skewed, 1000 bias corrected bootstrapped samples were used to calculate 95% confidence intervals for each regression parameter [19]. A predictor was considered to be significant if the 95% confidence intervals did not overlap with zero.

### Ethical considerations

Institutional Review Bord (IRB) approval for the study was obtained from King Saud University (KSU), reference number KSU-HE-21-231.

## Results

### Demographics

A total of 475 responses were collected. Majority of sample was female (68.8%); aged 18-29 years (30.1%); married (62.1%); held bachelor’s degree (55.4%); earned SAR 10,001-20,000 (32.8%); and public employee (34.7%). A detailed description of the demographics of the sample is shown in Table 1.

**Table 1:** Demographic characteristics of the sample (*N =* 475)

Characteristic	N	%
Gender		
Male	148	31.2
Female	327	68.8
Age		
18-29	143	30.1
30-39	124	26.1
40-49	116	24.4
≥ 50	92	19.4
Marital Status		
Single	148	31.2
Married	295	62.1
Other	32	6.7
Region		
Central	310	65.3
Eastern	95	20.0
Western	28	5.9
Southern	11	2.3
Northern	31	6.5
Education		
High school	88	18.5
2-year Diploma	67	14.1
Bachelor	263	55.4
Master	44	9.3
PhD	13	2.7
Income		
≤ 5000 SAR	152	32.0
5001-10,000 SAR	128	26.9
10,001-20,000 SAR	156	32.8
20,001-30,000 SAR	29	6.1
> 30,000 SAR	10	2.1
Occupation		
Public employee	165	34.7
Private employee	109	22.9
Unemployed	201	42.3
Type of healthcare Provider		
Public	237	49.9
Private	238	50.1
Satisfaction with public health services		
Very satisfied	75	15.8
Satisfied	183	38.5
Neutral	134	28.2
Unsatisfied	69	14.5
Very unsatisfied	14	2.9

### Willingness to pay (WTP)

**Fig. 1:**
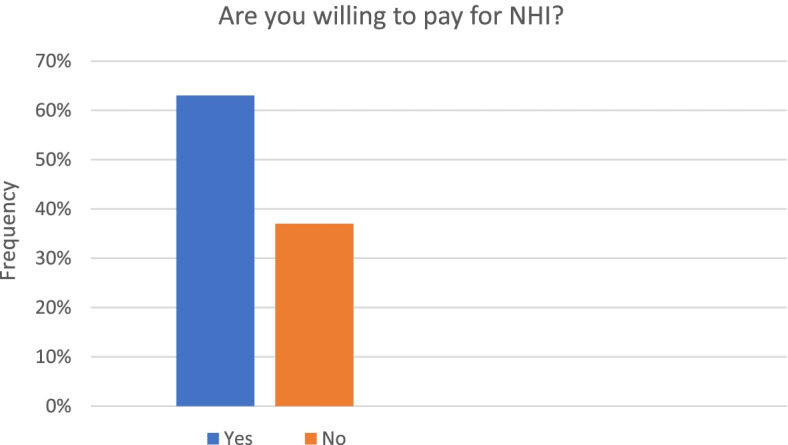
Frequencies of individuals willing and not willing to pay for NHI (*N =* 475)

The number of individuals who was willing to pay for NHI was higher than those who were not willing to pay (62.9, 95% CI = 58.4-67.3%) vs (37.1, 95% CI = 32.7-41.6%) [Fig. 1]. A binomial test found this difference was statistically significant (*p <* 0.001).

A multiple binary logistic regression was used to test the association between willingness to pay (WTP) and demographics, type of usual health care provider, and satisfaction with the healthcare services. As shown in Table 2, there was a significant association between the type of usual healthcare provider and the likelihood of paying for NHI (OR = 3.129, 95% CI = 1.943-5.039, *p <* 0.001). It seems that individuals using public health services were more likely to contribute and pay for NHI than those using private health services. Also, the analysis found a significant association between satisfaction with healthcare services and the likelihood of paying for NHI (OR = 14.305, 95% CI = 3.240-63.153, *p <* 0.001). The results show individuals who were very satisfied with the healthcare services were more likely to pay to NHI.

**Table 2:** Logistic Regression (*N =* 475)

Characteristic	OR	OR 95% CI	*P* value	
Gender				
Male (*n =* 89)	0.947	0.539	1.665	0.85
Female (ref) (*n =* 210)				
Age				
18-29 (*n =* 80)	1.415	0.618	3.241	0.41
30-39 (*n =* 64)	1.790	0.917	3.492	0.09
40-49 (*n =* 90)	0.636	0.311	1.300	0.21
≥ 50 (ref) (*n =* 65)				
Marital Status				
Single (*n =* 79)	1.156	0.407	3.285	0.79
Married (*n =* 198)	1.031	0.426	2.495	0.95
Other (ref) (*n =* 22)				
Region				
Central (*n =* 202)	1.062	0.449	2.512	0.89
Eastern (*n =* 55)	1.730	0.674	4.441	0.25
Western (*n =* 16)	0.853	0.268	2.713	0.79
Southern (*n =* 9)	0.172	0.022	1.362	0.10
Northern (ref) (*n =* 17)				
Education				
High school (*n =* 57)	7.189	0.724	71.423	0.09
2-year Diploma (*n =* 44)	5.993	0.588	61.188	0.13
Bachelor’s (*n =* 160)	9.249	0.977	87.517	0.05
Master’s (*n =* 26)	8.363	0.849	82.403	0.07
PhD (ref) (*n =* 12)				
Income				
≤ 5000 SAR (*n =* 85)	2.244	0.394	12.776	0.36
5001-10,000 SAR (*n =* 112)	1.987	0.364	10.835	0.43
10,001-20,000 SAR (*n =* 73)	0.995	0.184	5.397	1.00
20,001-30,000 SAR (*n =* 21)	2.584	0.389	17.177	0.33
> 30,000 SAR (ref) (*n =* 8)				
Occupation				
Public employee (*n =* 106)	1.331	0.716	2.473	0.37
Private employee (*n =* 66)	1.586	0.839	2.998	0.16
Unemployed (ref) (*n =* 127)				
Type of healthcare Provider				
Public (*n =* 127)	**3.129**	**1.943**	**5.039**	**< 0.001**
Private (ref) (*n =* 172)				
Satisfaction with public health services				
Very satisfied (*n =* 52)	**14.305**	**3.240**	**63.153**	**< 0.001**
Satisfied (*n =* 125)	2.196	0.976	4.942	0.06
Neutral (*n =* 78)	1.955	0.968	3.949	0.06
Unsatisfied (*n =* 40)	0.982	0.501	1.927	0.96
Very unsatisfied (ref) (*n =* 4)				

**Fig. 2:**
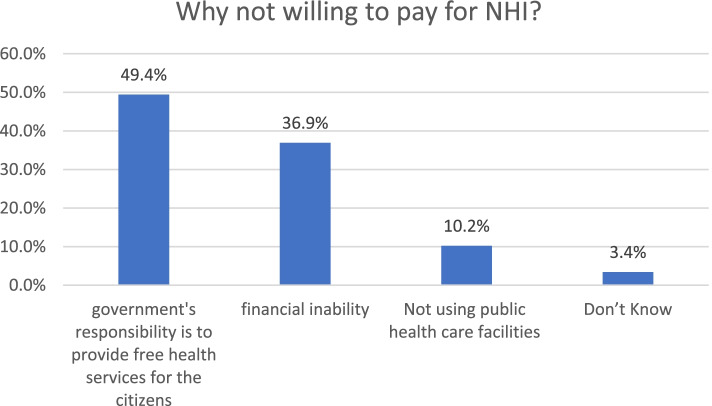
Reasons for unwillingness to pay for NHI (*N =* 176)

The reasons for not being willing to pay for NHI are shown in Fig. 2. Almost half of unwilling individuals (49.4%) indicated that they believed it was the government’s responsibility to provide free health services for the citizens. Approximately one-third (36.9%) stated financial inability as the reason for their unwillingness to pay for NHI, of which 57% had monthly income less than 5000 SAR; and 53% were unemployed [Additional file 2]. More details on income level and occupation for financially unable respondents are available in Additional file 2. Not using public healthcare facilities was the reason given by 10.2% of the unwilling respondents. The remaining 3.4% did not provide a reason.

### Factors affecting the amount of money individuals were willing to pay for NHI

**Fig. 3:**
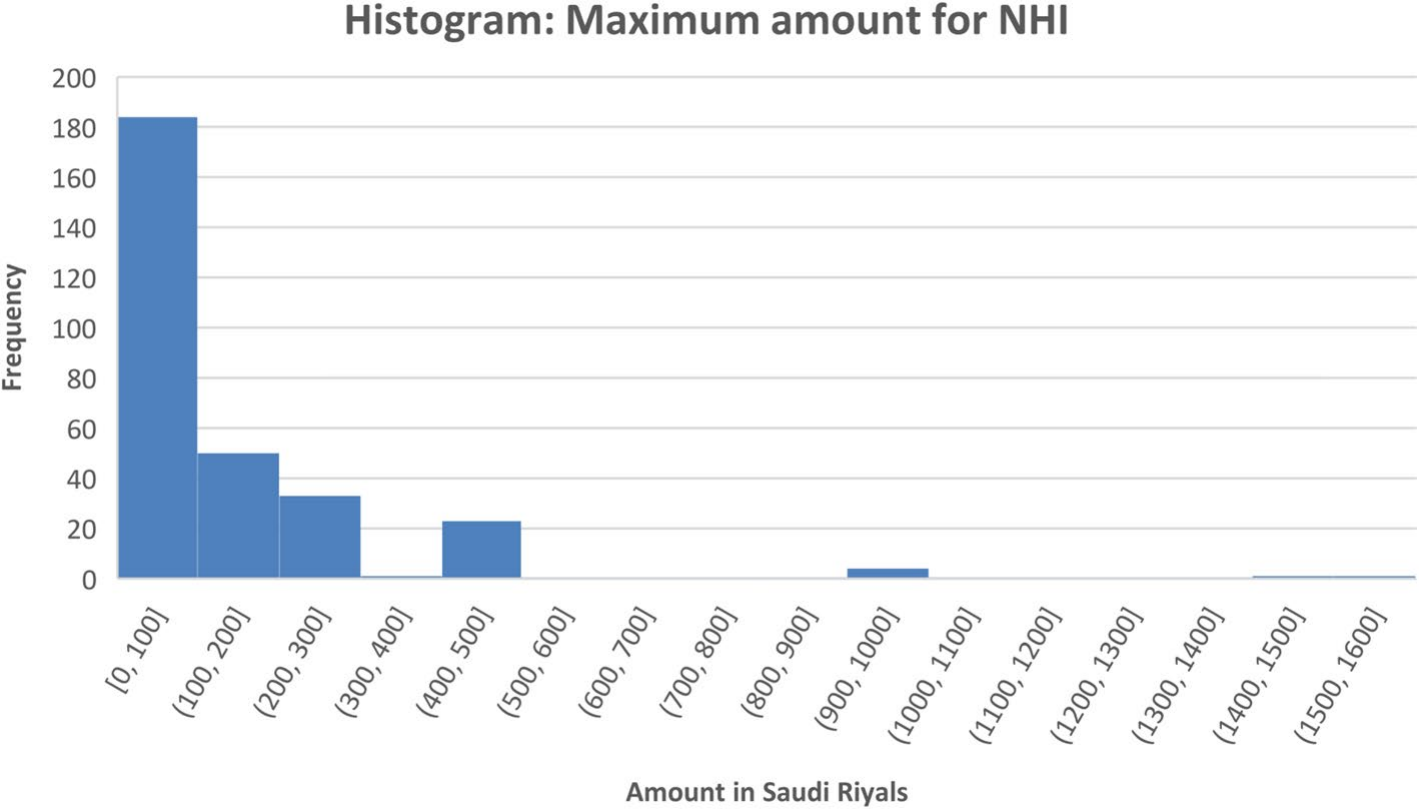
Distribution of maximum amount of money individuals are willing to pay for NHI (*N =* 299)

In our sample, 62.9% participants were willing to pay for NHI. The median amount of money the people were willing to pay as a monthly contribution for NHI was 100 SAR (26.5 USD) with the average being 152 SAR (40 USD) [Fig. 3]. As shown in Fig. 3, the majority of the respondents were willing to pay 100 SAR (26.5 USD) or less.

A multiple linear regression was conducted to test the association between the maximum amount of monthly contribution and several factors such as demographics, type of usual health care provider, and satisfaction with the healthcare services. Table 3 shows the results of this analysis. The overall model was statistically significant, *F* (25, 271) = 2.443, *p <* 0.001, and the R^2^ indicated a medium effect size based on Cohen’s (1992) guidelines (R^2^ = 0.184). The results show a significant association between age and the maximum amount the participants were willing to pay. Specifically, 30–39-year-olds were willing to pay more for NHI compared to participants aged 50 or older (ß = 103.55, 95% CI = 26.27- 199.29). In addition, the results show a significant relationship between region and the maximum amount of money people were willing to pay (central > northern) (ß = 70.71,95% CI = 2.14- 138.58). Lastly, people with masters’ degrees were willing to pay more for NHI compared to the participants with PhDs (ß = 227.46, 95% CI = 81.59- 399.28).

**Table 3:** Linear Regression (*N =* 299)

Characteristic	B	95% CI	
Gender			
Male	26.31	−26.94	87.76
Female (ref)			
Age			
**18-29**	0.53	− 113.74	81.06
30-39	**103.55**	**26.27**	**199.29**
40-49	−17.37	−83.23	43.57
≥ 50 (ref)			
Marital Status			
Single	−30.54	−188.62	104.92
Married	−95.39	−244.63	0.33
Other (ref)			
Region			
Central	**70.71**	**2.14**	**138.58**
Eastern	29.17	−53.70	111.02
Western	54.39	−84.41	170.36
Southern	−1.52	−86.68	77.05
Northern (ref)			
Education			
High school	84.78	−17.61	190.19
2-year Diploma	66.75	−35.78	189.53
Bachelor’s	88.46	−16.70	194.25
Master’s	**227.46**	**81.59**	**399.28**
PhD (ref)			
Income			
≤ 5000 SAR	38.28	−93.62	200.54
5001-10,000 SAR	6.10	− 122.82	147.97
10,001-20,000 SAR	73.40	−59.42	205.55
20,001-30,000 SAR	128.58	−21.02	275.74
> 30,000 SAR (ref)			
Occupation			
Public employee	26.79	−37.98	90.54
Private employee	−5.90	−74.96	60.77
Unemployed (ref)			
Type of healthcare Provider			
Public	−29.16	−81.69	16.71
Private (ref)			
Satisfaction with public health services			
Very satisfied	−61.30	− 193.66	51.57
Satisfied	−49.10	− 173.06	45.84
Neutral	−34.34	− 163.44	66.18
Unsatisfied	−37.27	−175.27	82.56
Very unsatisfied (ref)			

## Discussion

This study explored the willingness of the citizens of Saudi Arabia to pay for a contributory National Health Insurance. The results indicate that majority of Saudi citizens were willing to pay a monthly contribution for the National Health Insurance (NHI), if implemented, in return for a sustainable and improved quality of public healthcare services. This finding is consistent with previous studies that examined people’s willingness to pay for NHI in Jeddah city in Saudi Arabia [16,17]. Our study provides evidence that citizens from other regions were also in favor of a NHI. The type of usual source of healthcare and satisfaction with the current healthcare services were found to have significant impact on the willingness to pay for NHI. People who usually used private health facilities were less willing to pay for an NHI. This was probably because they already had a private health insurance through their employer, so they did not see the need for a NHI. In Saudi Arabia, the employers in the private sector are required by law to provide their employees with health insurance. On the other hand, people who usually used public health facilities were more willing to pay for an NHI. This was probably because they wanted to expand their choices and access private providers. Saudi citizens have always perceived private hospital services as being of a better quality than those in public hospitals [20]. Despite major investment in the public healthcare sector in Saudi Arabia by the government, many people remain dissatisfied with the availability and quality of care at publicly-run hospitals and clinics [9]. The free model currently used in the public sector has led to longer waiting time and the overutilization of emergency departments [10]. Increasing the involvement of the private sector through the NHI is an important approach that government is taking to decrease dependence on public funds and to improve the quality of care provided for NHI members. In addition to the type of usual source of care, the level of satisfaction with current health services was found to be a significant indicator of willingness to pay. People who were very satisfied with public services were more willing to pay for NHI. This finding is consistent with a previous study that found that the respondents who were satisfied with the quality of public healthcare services were more willing to pay for NHI than those who were not [16]. This is not surprising as people tend to pay for things they perceive as valuable and satisfactory and they believe their contribution is worthwhile. The hypothetical scenario presented to the respondents promised that the new NHI would help to sustain the level of health services currently provided, improve its quality, and expand access to private sector care.

The median amount that people were willing to pay as a monthly contribution for NHI was 100 SAR (26.5 USD) with the average being 152 SAR (40 USD). The rates currently applied in the private health insurance (citizens and non-citizens) fall in four categories, the highest with an average monthly rate of 167 Sar (44 USD), and the lowest at 71 SAR (19 USD) [21]. So, it seems that the amount of money the respondents were willing to pay falls within the range of the rates currently used in the private insurance market. Age was found to affect the maximum amount of money the individuals were willing to pay for NHI. It seems that young individuals were more likely to pay more money for NHI compared to older ones. This is consistent with other studies which found that age was a significant factor regarding the level of payment [22–25]. This is probably because the older people get, the more obligations they face which require dividing their limited resources carefully, while the younger people might have fewer financial responsibilities. In addition, education was found to affect the maximum amount of money the individuals were willing to pay for NHI. It seems that individuals with masters degree were more likely to pay more money for NHI compared to individuals with PhDs. This is contrary to previous research that found higher education level was associated with higher willingness to pay for health insurance [12, 26–28]. Lastly, the individuals’ place of residence was was found to affect the maximum amount of money the individuals were willing to pay for NHI. It seems that people from the central region were willing to pay more money for NHI than people from the northern region. This is probability because the central region has more urban areas in addition to the the capital city of Riyadh, where healthcare facilities are often more advanced, and typically provide all levels of care including specialist healthcare services, as compared with those in rural areas in the northern region. Previous research found people residing in urban areas were more willing to pay for health insurance [16, 29].

Almost one third of the study sample were unwilling to contribute to a national health insurance. Several reasons were provided. Almost half of these respondents stated that they believed it was the government’s responsibility to provide free health services for the citizens. This is not surprising as most Saudi citizens consider healthcare to be a right and are accustomed to the free service model. Recently, however, the government has realized that this model is unsustainable in the long run. In spite of an increased budget allocation for free public health services, the actual average expenditure per capita is expected to decrease [21]. This is because the population is rapidly growing and oil prices, from which most of the government revenues come, are fluctuating. Free healthcare has led to several problems such as overutilization and the abuse of services in addition to long waiting times and dissatisfaction with the quality of the services. Consequently, Saudi decision-makers are seeking to have a sustainable health system through the introduction of a contributory national health insurance. Other reasons for refusing to pay for NHI include some of the respondents not using public health services, and financial inability. More than half of the respondents who stated financial inability as their reason for not being willing to pay were unemployed or had monthly income less than 5000 SAR [Additional file 2], which may explain the lack of the means to contribute to a health insurance. Future research is recommended to explore the perceptions and barriers to implementing a national health insurance from the providers’ point of view.

One of the limitations of the contingent valuation technique is based on whether it adequately measures people’s willingness to pay for commodities which are not typically traded in the marketplace [30]. Contingent valuation assumes that individuals understand the system service in question and will express their preferences in the contingent market just as they would in a real one. However, most people are unfamiliar with placing monetary values on services that are not typically traded in the marketplace. Therefore, they may not have had a suitable basis for expressing their true value. It is also suggested that people place a different value on a good in a hypothetical situation compared to an actual situation [30]. Nevertheless, this study provides an exploration at the national level about the public’s willingness to pay for NHI, which should lead to further studies on the subject.

## Conclusion

This study provided some evidence that more people were willing to pay for NHI than those who declined. The factors that appeared to influence the willingness to pay and the amount of monthly payment included the type of usual source of care, satisfaction with current public services, age, and income. Individuals who frequently used public health services and were very satisfied with these services were more willing to pay for NHI. Younger population, those with master’s degree, and from the central region were willing to pay more amount of money for NHI. These results could help policy makers shape their decisions and anticipate problems that may arise with NHI implementation.

## Supplementary Information

**Additional file 1.**

**Additional file 2.**

## Data Availability

The dataset used was uploaded with the paper submission as a supplementary file.

## Abbreviations

NHI: National Health Insurance
WTP: Willingness to pay
MoH: Ministry of Health
IRB: Institutional Review Bord
KSU: King Saud University
