# Correction: Willingness to pay for a National Health Insurance (NHI) in Saudi Arabia: a cross-sectional study

**DOI:** 10.1186/s12889-023-16089-6

**Published:** 2023-06-21

**Authors:** Abeer Alharbi

**Affiliations:** Health Administration Department, Business Administration College, King Saud University, Riyadh, Saudi Arabia

**Correction: BMC Public Health 22, 951 (2022)**

**https://doi.org/10.1186/s12889-022-13353-z**

In the original publication [1] the full datasets were not available as stated in the data availability section. The full datasets are available in this correction article.

## Supplementary Information

**Additional file 1: Supplementary file 1.** Full datasets.
