# Supplementary material for: Willingness to pay for a National Health Insurance (NHI) in Saudi Arabia: a cross-sectional study
Source: BMC Public Health. 2022 May 12;22:951. doi: 10.1186/s12889-022-13353-z (PMC9103041; doi:10.1186/s12889-022-13353-z)
Supplement: Supplementary file 1 — Additional file 1. [file 12889_2022_13353_MOESM1_ESM.docx]

**Willingness to Pay for a National Health Insurance (NHI) in Saudi Arabia: A Cross-sectional Study**

**Authors**:

Abeer Alharbi, PhD, Health Administration Department, Business Administration College, King Saud University, Riyad, Saudi Arabia. Email: [aalharbi15@ksu.edu.sa](mailto:aalharbi15@ksu.edu.sa) (corresponding author)

**Appendix 1**

**Willingness to Pay (WTP) Contingent Valuation Survey**

Please read the following before answering the survey questions:

Currently, in the Kingdom of Saudi Arabia, public health care services are provided free of charge to all citizens through the government funding. Because these services are free, patients often face long waiting times before consulting a specialist or a consultant. The demand for public healthcare services is growing, but resources such as hospital bed capacity, doctors, and nurses may still be insufficient. Due to the increasing cost of providing health care, the government may not be able to cover all health care costs from its own resources in the future.

Imagine a hypothetical scenario where the government establishes a system (National Health Insurance) in order to ensure the sustainability and raise the quality of existing public health care services and citizens will be required to pay regular contributions to it. The contributions will complement the government's health budget to meet increased costs. Health care services that are currently available will continue to be available and free of charge. To increase the availability of services, this system allows the participation of the private sector in the provision of health services. The contribution will be similar to insurance premiums, with no refunds for those who do not need to use health services.

- Are you willing to contribute to the national health insurance system and pay a monthly health insurance premium in exchange for ensuring the sustainability and raising the quality of the current public health care services? – Yes – No
- If Yes - What is the highest amount (in Saudi Riyals) you would be willing to pay as a monthly health insurance premium in exchange for a National Health Insurance?
- If No - Identify the main reason why you are unwilling to pay for a National Health Insurance. Please choose the most important reason from the list below
- Do not use public healthcare services
- financially incapable
- The government's responsibility is to provide free health services
- I don’t know
