# Supplementary material for: Willingness to pay for a National Health Insurance (NHI) in Saudi Arabia: a cross-sectional study
Source: BMC Public Health. 2022 May 12;22:951. doi: 10.1186/s12889-022-13353-z (PMC9103041; doi:10.1186/s12889-022-13353-z)
Supplement: Supplementary file 2 — Additional file 2. [file 12889_2022_13353_MOESM2_ESM.docx]

**Willingness to Pay for a National Health Insurance (NHI) in Saudi Arabia: A Cross-sectional Study**

**Authors**:

Abeer Alharbi, PhD, Health Administration Department, Business Administration College, King Saud University, Riyad, Saudi Arabia. Email: [aalharbi15@ksu.edu.sa](mailto:aalharbi15@ksu.edu.sa) (corresponding author)

**Appendix 2**

Table: Frequencies of income level and employment status for those stated financial inability as their reason for not willing to pay for NHI

| Variables | | I am not willing to pay for NHI because I am financially incapable |
| --- | --- | --- |
| Income | ≤5000 SAR | 57% |
|  | 5001-10000 SAR | 31% |
|  | 10001-20000 SAR | 11% |
|  | 20001-30000 SAR | 1% |
|  | >30000 SAR | 0% |
| Occupation | Public employee | 28% |
|  | Private employee | 19% |
|  | Unemployed | 53% |
