# Supplementary material for: Correction: Willingness to pay for a National Health Insurance (NHI) in Saudi Arabia: a cross-sectional study
Source: BMC Public Health. 2023 Jun 21;23:1204. doi: 10.1186/s12889-023-16089-6 (PMC10286399; doi:10.1186/s12889-023-16089-6)
Supplement: Supplementary file 1 — Additional file 1: Supplementary file 1. Full datasets. [file 12889_2023_16089_MOESM1_ESM.zip › Survey questions with codes.pdf]

SURVEY QUESTIONS AND THEIR CODES IN THE EXCEL SHEET

Yes = 1 / No = 2	**A1**	**Are you willing to pay for NHI?**
statements	**A2**	**Reasons for not willing to pay**
continuous number	**A3**	**Amount of money in Saudi Riyals (continuous) willing to pay for NHI**
public = 1 / private = 2	**A4**	**Type of Healthcare Provider**
very satisfied = 5 / satisfied = 4 / neutral = 3 / unsatisfied = 2 / very unsatisfied = 1	**A5**	**Satisfaction level with health services**
male = 1 / female = 2	**A6**	**Gender**
18-29=1 /30-39=2 /40-49=3 />=50=4	**A7**	**Age**
married = 1 / single = 2 / other = 3	**A8**	**Marital statue**
Public employee = 1 / private employee = 2 / unemployed = 3	**A9**	**Occupation**
high school = 1 / 2-year diploma = 2 / bachelor = 3 / master = 4 / PhD = 5	**A10**	**Education**
less than 5000=1 /5000-10000=2 /10001-20000=3 /20001-30000=4 /30000 or more=5	**A11**	**Income**
central = 1 / eastern = 2 / western = 3 / southern = 4 / northern = 5	**A12**	**Region**

- (**A1**) Are you willing to contribute to the national health insurance system and pay a monthly health insurance premium in exchange for ensuring the sustainability and raising the quality of the current public health care services? – Yes – No

هل أنت على استعداد للمساهمة في نظام التأمين الصحي الوطني ودفع قسط تأمين صحي شهري مقابل ضمان استدامة ورفع جودة خدمات الرعاية الصحية العامة الحالية؟ نعم \ لا

- (**A2**) If No - Identify the main reason why you are unwilling to pay for a National Health Insurance. Please choose the most important reason from the list below:

  - Do not use public healthcare services
  - لا استخدم خدمات الرعاية الصحية العامة
  - financially incapable
  - غير مقتدر ماديا

- The government's responsibility is to provide free health services
- مسؤولية الحكومة توفير خدمات صحية مجانية
- I don’t know
- لا ادري

- (**A3**) If Yes - What is the highest amount (in Saudi Riyals) you would be willing to pay as a monthly health insurance premium in exchange for a National Health Insurance?

- (**A4**) Which of the following health care services are you most often used?
- – Public / Private
  - أي من خدمات الرعاية الصحية التالية تستخدم في الغالب:
- حكومي\خاص

- (**A5**) How satisfied are you with general healthcare services? – Likert scale
  - ما مدى رضاك عن خدمات الرعاية الصحية العامة
- راضي جدا very satisfied
- راضي satisfied
- محايد neutral
- غير راضي not satisfied
- غير راضي جدا very not satisfied

- **(A6) Gender: Male, Female**
- الجنس: ذكر / انثى
-
- **(A7) Age: 18-29, 30-39,40-49, >=50**
- العمر: 18-29 / 30-39 / 40-49 / 50 وأكبر
-
- **(A8) Marital Status: Single, Married, other.**
- الحالة الاجتماعية: اعزب / متزوج / أخرى
-

- (A9) **Occupation: Government employee, private employee, unemployed**
- الحالة الوظيفية: موظف حكومي / موظف خاص / غير موظف
-
- **(A10) Education: high school / 2-year diploma/ bachelor / master/ PhD**
- المستوى التعليمي: ثانوي / دبلوم / بكالوريس / ماجستير / دكتوراه
-
- **(A11) Income: less than 5000/5000-10000 /10001-20000 /20001-30000/>=30000**
- الدخل: اقل من 5000 / 5000-10,000 / 10,001-20,000 / 20,001-30,000 / اكثر من 30,000
-
- **(A12) Region: central / eastern / western / southern / northern**
- المنطقة: الوسطى، الشرقية، الغربية، الجنةبية، الشمالية
- **(A13) type of region: City, county, town**
- نوع المنطقة: مدينة، محافظة، هجرة
